# Vanadium Chemical Compounds Forms in Wastes of Vanadium Pentoxide Production

**DOI:** 10.3390/ma13214889

**Published:** 2020-10-30

**Authors:** Anton Volkov, Ulyana Kologrieva, Anatoly Kovalev, Dmitry Wainstein, Vladimir Vakhrushev

**Affiliations:** 1 State Scientific Centre I.P. Bardin Central Research Institute for Ferrous Metallurgy, 23/9 bdg. 2, Radio str., 105005 Moscow, Russia; rhenium@list.ru (A.V.); ufowka@mail.ru (U.K.); a_kovalev@sprg.ru (A.K.); gareq1211@gmail.com (V.V.); 2 Surface Phenomena Researches Group, Staropimenovskiy Lane, 6, bdg.1, app. 4, 127006 Moscow, Russia

**Keywords:** vanadium, vanadium sludge, chemical composition, oxidation degree, phase composition, microstructure, degree of extraction, waste treatment

## Abstract

A big amount of solid wastes or dump sludges is generated after leaching vanadium (V) from a roasted mixture. As the vanadium content in these tailings is comparable to its concentration in traditional vanadium sources such as titanomagnetite ores or a vanadium converter slag, these wastes could be recycled to extract additional vanadium. Therefore, this research was aimed on studies of vanadium-containing sludges resulting from hydrometallurgical production of vanadium pentoxide to find an optimal technology for V extraction. The material composition of industrial and synthetic sludge samples was studied by X-ray fluorescence analysis (XRF), X-ray diffraction (XRD), secondary ions mass spectroscopy (SIMS), and X-ray photoelectron spectroscopy (XPS, ESCA). The paper demonstrates the presence of vanadium in sludges, not only in spinels in 3+ oxidation degree, but also in other compounds containing V^4+^ and V^5+^. It was found that vanadium substitutes a set of elements in minerals except spinel. The dependence between the content of insoluble vanadium compounds and V oxidation degree was determined.

## 1. Introduction

Despite the relatively low content in the earth crust, vanadium now finds a wide range of applications. It is used in medicine, chemical industries including organic synthesis and fabrication of polymer materials, aerospace industry, nuclear power generation, glass, porcelain and earthenware industry, etc. [1,2,3,4,5,6,7,8,9,10], but most of the vanadium (about 80%) is consumed by ferrous metallurgy: It is a part of more than 250 steel and cast iron grades. Vanadium is a very important alloying element for the production of pipes for high pressure gas and oil pipelines [11,12,13].

The key raw materials for vanadium extraction are titanomagnetite ores (~88% of total V production). It could be extracted also from coal, utilized catalysts, etc. [14,15,16,17,18,19,20,21,22,23,24].

Technical vanadium pentoxide is fabricated in Russia from a converter vanadium slag from the EVRAZ NTMK metallurgical plant, using calcareous-sulphuric technology that includes the following stages: Crushing of the slag and mixing with limestone; calcification roasting; leaching of V compounds by sulphuric acid; hydrolytic sedimentation of vanadium pentoxide from the solution; fabrication of molten vanadium pentoxide [13,25].

Vanadium in the vanadium converter slag is presented in V^3+^, V^4+^, V^5+^ oxidation degrees [26,27]. Slag roasting is aimed on the oxidation of V^3+^ and V^4+^ to V^5+^, which is represented as acid-soluble compounds that pass into the solution during further leaching.

However, in real industrial conditions a big amount of solid wastes or dump sludges is produced after leaching V from a roasted mixture [25]. Dump sludges typically have the following compositions: 1.5–4.5 wt% V_2_O_5_; 40–55 wt% Fe_2_O_3_; 5–12 wt% MnO; 6–12 wt% TiO_2_; 2.5–5.5 wt% Cr_2_O_3_; 13–18 wt% SiO_2_; up to 15 wt% CaO [26,28]. The V content in these wastes is bigger than the titanomagnetite ore and concentrate from the EVRAZ KGOK ore-processing plant, which is used as primary raw materials in the chain of vanadium-containing converter slag fabrication. Therefore, the tailings of hydrometallurgical production of the vanadium pentoxide could be estimated as a prospective technogeneous raw material for vanadium extraction.

Therefore, this research was aimed on the determination of vanadium compound forms in the vanadium sludge to find an optimal technology for V extraction.

## 2. Materials and Methods

### 2.1. Methods

#### 2.1.1. X-ray Fluorescence Analysis

The chemical composition of vanadium slags listed in Table 1 was determined using the AXIOSmax Advanced (PANalytical, Almelo, Netherlands) X-ray fluorescence (XRF) spectrometer, by the method described in [29].

#### 2.1.2. Synthesis of Model Vanadium Containing Sludges

Model synthetic sludges were fabricated in the following way. The slag samples milled to a 0.2 mm fraction were roasted with CaCO_3_ in the amount CaO/V_2_O_5_ = 0.42–0.45 in the muffle furnace at 850 °C during 2 h. Then, the two-stage leaching of the mixture after cooling and milling to a 0.2 mm fraction was performed in the following conditions. The first stage was carried out at heating to 60 °C, solid/liquid (S/L) relation = 1:4, pH of the solution = 2.5–2.7 during 60 min. After filtration and water washing of the leaching cake (S/L = 1:1), the second stage of leaching (acid re-extraction) was made: A filter cake was processed by a 3–4% H_2_SO_4_ solution at S/L = 1:2 and washed by water at S/L = 1:1. The leaching residual was correspondent to the dump sludge.

The chemical analysis of vanadium sludge was fulfilled also using the AXIOSmax Advanced (PANalytical, Almelo, Netherlands) X-ray fluorescence spectrometer. Taking into account the absence of certified reference standards of V sludges, the synthetic samples were used for the spectrometer calibration. For this purpose, a mixture of calcium sulphate and oxides of elements were presented in the sludges in various proportions. Substances of high and AR grades and the GBW 03109a Gypsum reference sample (approved by the State Bureau of Technical Supervision The People’s Republic of China) were used. Substances of Al_2_O_3_, TiO_2_, MgO, SiO_2_, Ca_3_(PO_4_)_2_ were baked at 950 °C during 2–3 h in a muffle furnace. Samples of Na_2_SO_4_, V_2_O_5_, Cr_2_O_3_, Fe_2_O_3_, MnO_2_ were dried at 110 °C during 8 h. Melting fluxes were dried in a baker during 3 h. Melting of samples of V sludges and synthetic calibration samples were made in platinum crucibles with the mixture FX-X65-2 lithium tetraborate 66%—lithium metaborate 34%, produced by Fluxana GmbH&Co.KG (Bedburg-Hau, Germany) in the melting furnace Eagon 2 (PANalytical, Almelo, the Netherlands).

The acid-soluble vanadium (V_2_O_5a.s._) concentration was determined by the redox titration method. The sludge sample with a mass of 20 g was dissolved in the 7% solution of H_2_SO_4_ at S/L = 1:10 with permanent agitating by an overhead stirrer during 30 min. The solution was filtered under a vacuum. The precipitate was washed by 50 mL of water. The volume of the filtered solution was determined using a volumetric cylinder. Aliquots for titration were taken from the solution.

Humidity of the V sludge samples was determined by drying during 2 h at 110 °C. Losses at roasting were determined using a muffle furnace after heating to 1050 °C during 2 h.

#### 2.1.3. X-ray Diffraction Analysis

X-ray diffraction analysis (XRD) of samples was performed on the analytical installation ARL 9900 Workstation (Thermo Fisher Scientific, Waltham, MA, USA) combining a XRF spectrometer with an upper tube and a θ–θ diffractometer. Samples for XRD analysis were prepared by the pressing of powder to be analyzed into the boric acid substrate.

#### 2.1.4. Secondary Ions Mass Spectroscopy

Elemental distribution and phase composition of the sludge samples were studied by the time-of-flight secondary ions mass spectroscopy (TOF SIMS) method using the TOF.SIMS5-100 (IONTOF GmbH, Münster, Germany) mass spectrometer.

#### 2.1.5. X-ray Photoelectron Spectroscopy

Vanadium oxidation degrees in sludges were determined by X-ray photoelectron spectroscopy (XPS) on ESCALAB Mk2 (VG, East Grinstead, UK) at a vacuum of 3 × 10^−8^ Pa. XPS measurements were fulfilled using an X-ray Al Kα—Mg Kα twin anode source (hν = 1486.6 eV, Au 4f3/2–5/2 full width at half maximum (FWHM) = 0.9 eV). Powder samples were used to analyze the averaged compositions. The powder was placed on the special sample holder using a conducting carbon ribbon. The sample charge was suppressed by a slow energy electron bombardment with energy of 30 eV. The fine structure of XPS lines was analyzed using the UNIFIT2007 [30] software. Binding energies were corrected based on the position of C 1s line used as an internal reference.

### 2.2. Materials

Vanadium sludges from EVRAZ Vanady Tula (Tula, Russia) and model synthetic sludges, made in laboratory conditions from EVRAZ NTMK (Nizhniy Tagil, Russia) vanadium-containing converter slags with compositions, are listed in Table 1 by the calcareous-sulphuric technology.

The chemical composition of synthetic (samples #1–#4) and industrial (samples #5, #6) V sludge samples is listed in Table 2. One can see that the V_2_O_5_ content in industrial samples is significantly bigger than in synthetic ones.

## 3. Results and Discussion

**Figure 1:**
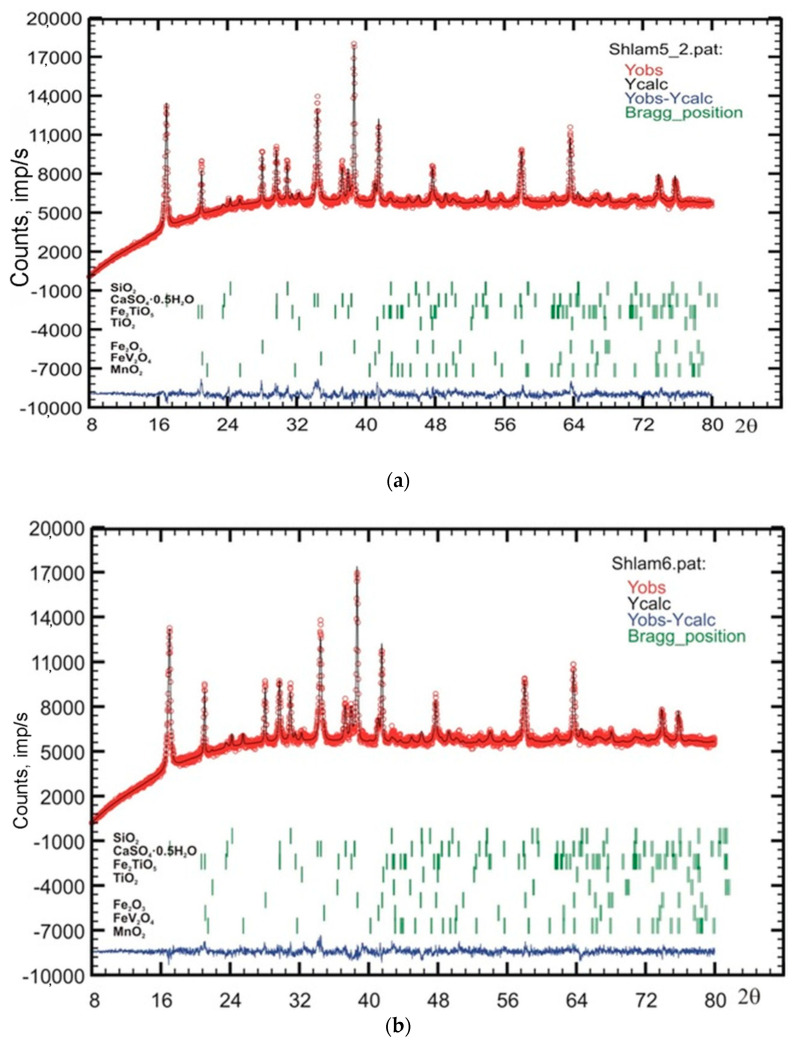
Experimental (red), theoretical (black), and differential (blue) diffractograms acquired from industrial sludge samples #5 (**a**) and #6 (**b**). Green dashes correspond to the positions of reference diffraction peaks of correspondent phases.

Figure 1 demonstrates the XRD pattern recorded from sample #1. Results of the XRD analysis presented in Table 3 demonstrate the presence of the following minerals in the V sludge: Hematite Fe_2_O_3_ (30.6–39.5%), bassanite CaSO_4_·0.5H_2_O (16.0–21.9%), solid solution of the pseudobrookite-armalcolite (Fe_0.5_Mg_0.5_)Ti_2_O_5_ type (13.5–21.4%, the structure varies in different samples). The silicate part is presented by grossular Ca_3_Al_2_Si_3_O_12_ (0.7–1.9%, absent in some samples) and quartz SiO_2_ (2.8–6.0%). Manganese is found in two minerals: Ramsdellite MnO_2_ (0.8–3.5%) and pyrochroite Mn(OH)_2_ (1.5% in one sample). Rutile TiO_2_ (1.1–2.5%) and spinel FeV_2_O_4_ (2.2–4.4%) were also found in sludge samples. The amorphous phase amount in the samples under study varied from 5 to 29%.

**Figure 2:**
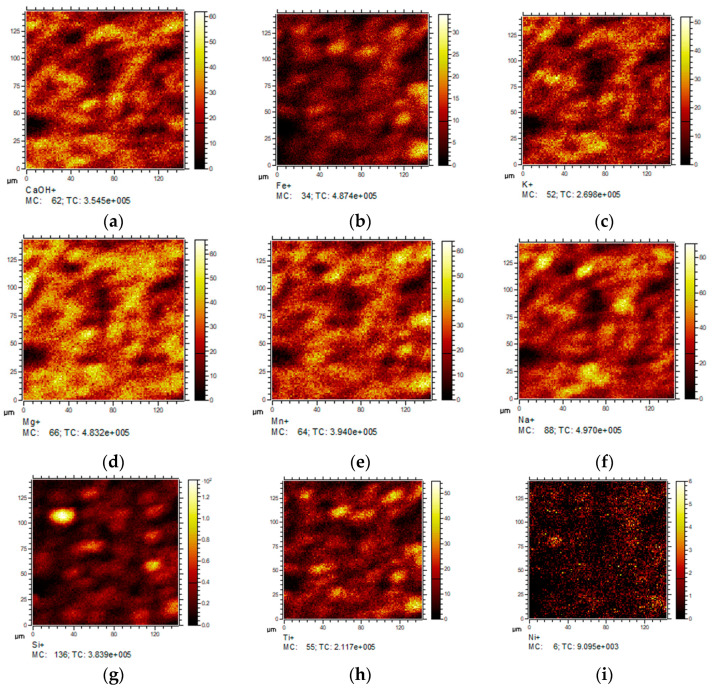

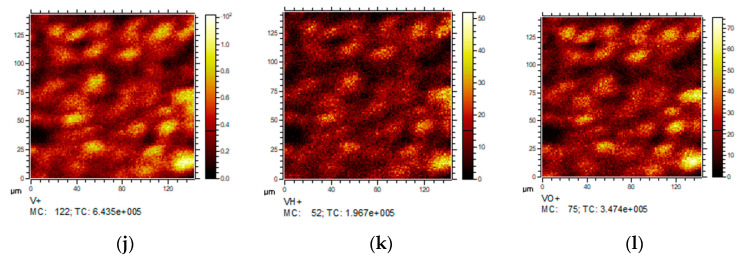
Secondary ions distribution maps in the sludge powder sample #5 (scanning SIMS): (**a**)—CaOH^+^; (**b**)—Fe^+^; (**c**)—K^+^; (**d**)—Mg^+^; (**e**)—Mn^+^; (**f**)—Na^+^; (**g**)—Si^+^; (**h**)—Ti^+^; (**i**)—Ni^+^; (**j**)—V^+^; (**k**)—VH^+^; (**l**)—VO^+^.

The elements distribution maps in the powder sludge sample #1 recorded using scanning TOF SIMS are presented in Figure 2. The powder particles have a diameter about 40 to 50 μm. The particles have a layered structure of the “core-shell” type. The compound of Ca, Mn, Fe, Al, Si, Ti, as well as carbon impurities in organic compounds are presented on the surface. Similar elemental distribution maps were obtained for sample #1, as in the other cases. It was found at the SIMS studies presented in Figure 2 that V is forming bonds mostly with Mn and Ti and in a lesser degree with Si. Other synthetic and industrial samples have similar qualitative regularities of elements correlations.

**Figure 3:**
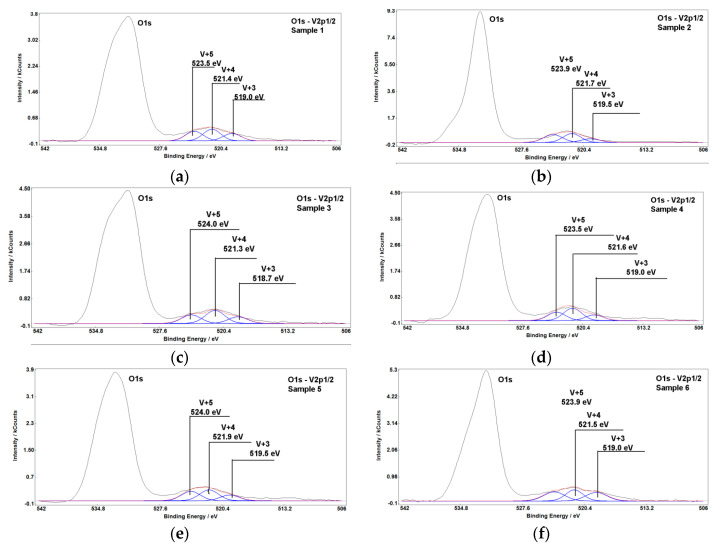
XPS O 1s—V 2p_1/2_ spectra from the surface of sludge samples #1 (**a**), #2 (**b**), #3 (**c**), #4 (**d**), #5 (**e**), #6 (**f**).

Figure 3 demonstrates the results of XPS analysis of V-containing sludge samples [27,31,32,33]. The layered structure of sludge particles and complex oxides on their surface leads to a very low intensity of the V2p_3/2_ XPS line. As such, the fine structure of a stronger V 2p_1/2_ line well defined on the spectra was analyzed in this research to determine the oxidation degrees of vanadium.

From Table 4, it can be seen that V is presented in the sludge in V^3+^, V^4+^, and V^5+^ oxidation degrees. V^3+^ is included in spinels of Me^2+^(Me^3+^)_2_O_4_ type (see Table 3), where Me^3+^ = V, Ti, Fe, Cr, Me^2+^ = Fe, Mn. V^4+^, V^5+^ could be found in an amorphous phase, as well as in Fe_0.5_Mg_0.5_Ti_2_O_5_, Fe_2_TiO_5_, and TiO_2_ where vanadium substitutes titanium. V^4+^ could be presented in the spinel of Me^4+^(Me^2+^)_2_O_4_ type where Me^4+^ = V, Ti, Mn, Me^2+^ = Fe, Mn. The decomposition of the spinel during baking and leaching leads to the formation of separate phases containing TiO_2_ (rutile and pseudobrookite-armalcolite solid solution) [18] found in the sludge in a significant amount. In this case, vanadium could reside in the sludge in a solid solution with TiO_2_ in pseudobrookite and/or rutile phases. This assumption is based on a close ionic radii of Ti^4+^ (0.75 Å [34]) and V^4+^ (0.72 Å [35]), therefore vanadium could easily substitute titanium in the oxide lattice.

The maximal concentration of insoluble vanadium (V_2_O_5_-V_2_O_5a.s._) is found in samples #2 and #5 possessing the maximal concentration V^3+^ while the lowest content of V^3+^ is observed in samples #3 and #4 with a minimal amount of insoluble vanadium. However, no correlation was found between the V^3+^ content and amount of the spinel as a main vanadium-containing phase in the sludge. Variations of the unit cell parameter α are indicating changes in the chemical composition of a spinel: Therefore, minimal α values in samples #2 and #3 correspond to the maximal content of Ti in probes, while maximal α values in samples #1, #5, and #6 are associated with marginal concentrations of Mn and Cr: Maximal in sample #1 and minimal in samples #5 and #6.

The maximal content of V^4+^ was found in samples #3 and #4 having a maximal amount of rutile (TiO_2_) excluded sample #1 with increased Ca content. At the same time, there is no correlation between the total Ti content and amount of rutile in the sludge. Therefore, based on experimental results we can conclude that V^4+^ partly substitutes titanium in rutile. This substitution is accompanied by a minimal quantity of insoluble vanadium (V_2_O_5_-V_2_O_5a.s._) in the probes.

Sample #1 with a maximal Ca content has an increased concentration of V^5+^, the lowest amount of the amorphous phase, and the presence of pseudobrookite-armalcolite Fe_0.5_Mg_0.5_Ti_2_O_5_ instead of armalcolite Fe_2_TiO_5_ is observed in other samples.

Thus, the amount of insoluble vanadium that forms in the sludge is controlled not only by the phase composition of samples and elemental composition of vanadium-containing minerals, but also by the oxidation degrees of V influencing on the recycling processability [26,36]. A typically higher amount of insoluble V^5+^ (V_2_O_5_-V_2_O_5a.s._) is linked with a higher concentration of V^3+^ but connections between V^3+^ and the amount of spinel that should hold it were not found. Samples with an increased content of rutile and vanadium in V^4+^ and V^5+^ oxidation degrees have a lower amount of insoluble form. Therefore, the extraction of V from dump vanadium sludges by hydrometallurgical methods will require the oxidation baking stage with the addition of calcium compounds for vanadium oxidation and its transformation to a soluble compound.

## 4. Conclusions

The studies of industrial vanadium sludges and laboratory samples with 1.33–3.67 wt% of V_2_O_5_ were performed. It was established that vanadium in sludges is presented in forms of V^3+^, V^4+^, and V^5+^ in spinel, rutile, and armalcolite. The paper demonstrates the dense connection of insoluble V forms content with the composition of vanadium-containing minerals and oxidation degrees of vanadium. Based on the obtained experimental data, we propose a further direction of research to improve the technology for extracting vanadium from sludges.

## Figures and Tables

**Table 1:** Chemical composition of vanadium (V) slags, wt%.

Slag No.	V_2_O_5_	MnO	SiO_2_	CaO	MgO	TiO_2_	Cr_2_O_3_	P_2_O_5_	Al_2_O_3_	Fe	S
1	24.3	14.98	13.1	3.98	1.63	9.95	4.20	0.049	1.66	23.2	0.013
2	23.6	13.73	11.2	2.17	3.03	12.91	4.08	0.036	2.41	24.0	0.006
3	20.7	11.05	10.0	1.68	4.12	11.27	3.47	0.092	3.52	29.1	0.005
4	21.4	11.99	17.2	2.76	2.95	8.40	3.58	0.063	4.29	22.4	0.013

**Table 2:** Chemical composition of V sludge samples, wt%.

Component	Sample					
	#1	#2	#3	#4	#5	#6
Na_2_O	0.11	n/d *	n/d	0.19	0.18	0.13
MgO	1.01	2.48	2.53	2.45	0.84	1.53
Al_2_O_3_	1.21	1.85	2.12	3.38	1.34	2.10
SiO_2_	13.09	9.09	8.46	16.81	11.0	11.20
P_2_O_5_	0.01	0.01	n/d	0.02	0.03	0.04
K_2_O	0.054	0.022	0.018	0.310	0.107	n/d
CaO	11.9	9.8	9.1	10.1	11.9	10.0
TiO_2_	9.7	12.2	11.25	8.8	7.5	7.69
V_2_O_5_	1.67	1.93	1.77	1.33	3.67	2.78
V_2_O_5a.s._	0.03	0.03	0.19	0.09	1.4	1.14
Cr_2_O_3_	4.26	4.07	3.93	3.81	3.34	3.02
MnO	9.90	7.70	6.94	7.37	6.64	6.68
Fe_2_O_3_	32.9	37.3	40.8	33.0	36.5	38.7
SO_3_	14.30	~12.3	~11.3	~11.3	15.1	4.90
LOI	11.25	n/d	n/d	n/d	n/d	11.3
Humidity	4.19	n/d	n/d	n/d	6.2	22.3

* Not defined.

**Table 3:** Phase composition of vanadium sludges.

Phase	Sample					
	#1	#2	#3	#4	#5	#6
bassanite (CaSO_4_·0.5H_2_O)	21.9	17.0	16.6	16.0	17.3	17.4
hematite (Fe_2_O_3_)	38.6	32.5	39.5	33.4	30.6	30.8
pseudobrookite-armalcolite (Fe_0.5_Mg_0.5_Ti_2_O_5_)	16.2	-	-	-	-	-
armalcolite (Fe_2_TiO_5_)	-	21.4	18.8	14.5	13.5	16.5
spinel (FeV_2_O_4_)	4.0	3.8	2.2	2.6	3.0	4.4
Spinel unit cell parameter, Å	8.457	8.433	8.428	8.437	8.452	8.451
quartz (SiO_2_)	5.6	3.1	2.9	2.8	4.5	6.0
Rutile (TiO_2_)	2.1	1.1	1.9	2.5	1.2	1.2
pyrochroite (Mn(OH)_2_)	1.5	-	-	-	-	-
ramsdellite MnO_2_	3.5	1.5	1.5	2.7	0.8	1.3
grossular Ca_3_Al_2_Si_3_O_12_	1.9	1.8	1.1	0.7	0.0	0.0
Amorphous phase	5	18	15	25	29	22

**Table 4:** Content of vanadium with different oxidation degrees in sludges.

Sample	Binding Energy, eV	Oxidation Degree	Fraction, %
#1	523.5	V^3+^	33.6
	521.4	V^4+^	40.5
	519.0	V^5+^	25.9
#2	523.9	V^3+^	39.9
	521.7	V^4+^	41.7
	519.5	V^5+^	18.4
#3	524.0	V^3+^	30.2
	521.3	V^4+^	46.2
	518.7	V^5+^	23.6
#4	523.5	V^3+^	31.7
	521.6	V^4+^	46.8
	519.0	V^5+^	21.5
#5	524.0	V^3+^	36.3
	521.9	V^4+^	41.6
	519.5	V^5+^	22.1
#6	523.9	V^3+^	34.0
	521.5	V^4+^	33.9
	519.0	V^5+^	32.1
